# Defining Frailty With the sWHI Tool in the SHARE Database: Predicting Falls and Fractures Occurrence Across Waves

**DOI:** 10.1093/geroni/igaf122.231

**Published:** 2025-12-31

**Authors:** Paolo Mazzola, Justin Brathwaite

**Affiliations:** University of Milano-Bicocca, Monza, Lombardia, Italy; University of Minnesota, Minneapolis, Minnesota, United States

## Abstract

**Introduction:**

Tools for assessing frailty can be used both in clinical settings and in community medicine. However, the practice of frailty assessment in community medicine is often limited by time constraints and workload reported by general practitioners.

**Methods:**

Accessing the Survey of Health, Ageing and Retirement in Europe (SHARE), we performed descriptive statistics of the study population belonging to Wave 8 interviews. Frailty was defined with 4 questions of the Simplified sWHI tool (range 0-5): 1) extent of limitation in daily activities (score 0-2); 2) frequency of feeling tired (0-1); 3) frequency of practicing activities that require a moderate amount of energy (0-1); 4) reported weight loss of > 4 kg (0-1). Subjects were frail for sWHI score>3, pre-frail for sWHI score 1 or 2, and non-frail for sWHI=0. Outcomes were hip fracture occurrence and falls occurrence in the last 6 months (evaluated at Wave 9 interviews).

**Results:**

The study population included 31,471 individuals (mean age 71.2±9.5 years): 10,242 were Non-frail, 15,435 were Pre-frail, and 5,139 were Frail. Multivariable logistic regression analyses, adjusted for age, sex, and comorbidity, showed that being pre-frail and frail increases significantly and independently the risk of hip fractures (OR 1.9 and 3.6, respectively ) and falls occurrence (OR 1.7 and 3.7, respectively) compared to being non-frail (p<.001).

**Conclusions:**

The sWHI tool is useful and quick for identifying frailty with 4 simple questions, and can be adopted by general practitioners to guide the implementation of prevention strategies against this syndrome.

